# Our Journal in 1886

**Published:** 1885-12-20

**Authors:** 


### EDITORIALS AND MISCELLANEOUS.

OUR JOURNAL IN 1886.—That our journal in the past has been very popular with the busy practitioner, especially in the South and West, we can truthfully say, and we have evidence that it is gaining in popularity, as we have already many new names coming in for the new year. We ask our present subscribers to speak a kind word for the RECORD among their medical acquaintances. Remember our liberal terms.

